# Factors influencing medical students’ approaches to learning in Qatar

**DOI:** 10.1186/s12909-022-03501-9

**Published:** 2022-06-09

**Authors:** Sheila S. Qureshi, Adam H. Larson, Venkat R. Vishnumolakala

**Affiliations:** 1 Weill Cornell Medicine-Qatar, P.O. Box 24144, Doha, Qatar; 2 Curtin University, Perth, Australia

**Keywords:** Qatar, Medical education, Students’ approaches to learning, R-SPQ-2F, Transnational education

## Abstract

**Background:**

This study investigated the relevance of the revised 2-factor study process questionnaire (R-SPQ-2F) for exploring medical students’ approaches to learning in Qatar and identify how factors like gender, age, educational attainment, and prior experience with health care influence students’ adoption of deep approaches to learning.

**Methods:**

The sample consisted of 108 medical students (44% male, 56% female) from all four years of medical school at Weill Cornell Medicine-Qatar (WCM-Q). Participants completed the 20-item R-SPQ-2F questionnaire to measure their learning approaches through a structural model contrasting deep and surface learning. Participants also completed a survey collecting demographic information.

**Results:**

Statistical analysis revealed significant differences in deep learning approaches across year levels for both men and women. Additionally, educational attainment played a significant role in students’ approaches to learning.

**Conclusions:**

Based on structural equation modeling, this cross-verification study supports the R-SPQ-2F instrument and offers additional evidence for its robustness and application to medical education. These findings may help educational and program leaders in Qatar better understand medical students’ learning approaches to enhance their pedagogical practices.

## Background

Medical educators aim to cultivate compassionate physicians who think critically about patient care [1]. Patients expect competent doctors who incorporate the latest developments in medical research into their practice. Medical educators have implemented innovative teaching strategies, such as problem-based learning (PBL), to develop students’ critical thinking, analytical reasoning, and metacognitive awareness [2]. However, these strategies contrast with common assessments in medical education that emphasize achievement on multiple-choice examinations. This draws attention to the design of learning and assessment tasks and how it relates to students’ approaches to learning.

### The 3P model of students’ approaches to learning

Researchers have investigated the role of students’ study behaviors in learning and academic performance for decades [3–7], and medical education is no exception [8–10]. Among the most influential models of how students approach learning tasks is Biggs's 3P model [5]. The 3P model illustrates how student characteristics and perceptions, teaching context, learning tasks, and learning outcomes interact to form an active system of teaching and learning. The 3 Ps—*presage*, *process*, and *product*—dynamically interact and influence one another. First, *presage* involves the elements in place before a student enters a learning context, and therefore is outside the teacher’s control. It includes characteristics like prior knowledge, ability, and personality, as well as contextual factors like subject area, instructional methods, modes of assessment and evaluation, and course structure [5].

Second, *process* refers to students’ motives and strategies, which together constitute an *approach to learning*. Students adopt deep or surface approaches to learning based on complex interactions between individual and institutional characteristics, and student perceptions of learning environments and tasks. For instance, when students adopt a surface approach to learning, they show instrumental motives and employ strategies aimed at limiting learning to the “bare essentials” they can easily memorize [5, 11]. Students who adopt a deep approach to learning hold intrinsic motives aimed at realizing learning in a subject and employ meaningful strategies to link prior learning to course content [5, 11, 12]. Yet, it is important to point out that approaches to learning are not inherent student attributes; rather, students can apply both deep and surface approaches depending on context. For example, both intrinsic interest in a subject and desire for achievement and social recognition can motivate student learning [5, 11].

Finally, *product* denotes the outcomes of learning, which Biggs [5, 11] describes as both performance measures such as grade point average (GPA) and examination scores, and achieving personal goals and satisfaction. Subsequent research has added more holistic graduate attributes, including self-reflection, lifelong learning, critical inquiry, and collaboration [12, 13]. The 3P model for teaching and learning underscores the notion that students’ choices to adopt surface or deep learning approaches are contextual, not essential. In this way, the model empowers teachers to design courses with learning tasks and assessments aimed at fostering motivation and strategies for deep learning. To effectively measure students’ approaches to learning, Biggs et al. [11] developed the revised 2-factor study process questionnaire (R-SPQ-2F).

### Students’ approaches to learning and medical education

Fostering deep learning approaches and encouraging inquiry and innovation are important topics in medical and allied health professional education [2]. Recent studies have explored how approach to learning relates to career preference, clinical experiences, progress testing and perceived stress, and PBL [14–17]. Several studies have statistically validated the R-SPQ-2F while exploring medical students’ approaches to learning. For instance, Vaughan [18] performed a confirmatory factor analysis to support the instrument’s reliability and validity in his study of Australian osteopathy students, as did Shaik et al. [19] in their study of the learning approaches of medical students in Saudi Arabia.

Studies suggest medical students prefer deep approaches to learning. For instance, Mansfield et al. [20] administered the R-SPQ-2F to students in Australia and found that medical students and applicants to medicine showed greater preference for deep learning than third-year science students. Similarly, Shaik et al. [19] surveyed 622 medical students at King Saud University using the R-SPQ-2F and identified strong preferences for deep learning. However, students with high GPAs and those who studied over five hours per day had the strongest preference. Tetik et al. [21] explored the relationship between students’ approaches to learning and curriculum at 3 medical schools in Turkey using hybrid, integrated, and PBL curricula, respectively. After administering the R-SPQ-2F to nearly 1,000 students, the authors discovered that the medical school using PBL experienced no decline in deep approach in the second year. From this, they concluded that PBL fosters deep approaches to learning because of the supportive, collegial nature of the PBL environment [21]. Likewise, Mogre and Amalba [17] examined students’ approaches to learning at a medical school in Ghana that uses PBL. They determined that students transition to deeper learning as they mature, suggesting that time in program and experience with PBL foster intrinsic motivation and deeper learning strategies. These studies from diverse geographical contexts stimulated our interest in medical students’ approaches to learning in Qatar.

### Research questions

In this paper, we surveyed medical students in Qatar to better understand the factors influencing their approaches to learning. The research questions addressed include:

- How well does the R-SPQ-2F fit the Qatari context?
- How do medical students in Qatar approach learning tasks?
- What student characteristics influence students’ approaches to learning?
- How does approach to learning interrelate with inquiry-based learning tasks like PBL?

Administering the R-SPQ-2F to medical students in Qatar further validates the instrument and provides valuable insights into the factors influencing students’ approaches to learning. Weill Cornell Medicine-Qatar (WCM-Q), the research setting, has revised its curriculum to feature individualized learning, research training, and greater use of PBL. However, multiple-choice assessments continue to dominate students’ experiences, as they must prepare for the United States Medical Licensing Examination (USMLE) Step 1, a critical standardized examination following the second year of medical school. Our aim was to explore students’ approaches to learning and how they relate to student characteristics and increased use of PBL in Qatar.

## Methods

### Setting

The study took place at Weill Cornell Medicine-Qatar (WCM-Q), an international branch campus founded in 2001 as a joint venture between Cornell University and the Qatar Foundation. WCM-Q was Qatar’s first medical school and the first American medical school established outside the United States [22]. It is a research-intensive, academic medical institution with the mission to “develop outstanding physicians, scientists, and future healthcare leaders; generate significant discoveries that transform healthcare; and promote population health through deeply-rooted community engagement” [22]. WCM-Q offers an integrated 6-year program leading to an American medical degree with a 2-year premedical program and 4-year medical program. It also offers a foundation program for qualifying students.

### Participants

Medical students aged 18 and older currently enrolled at WCM-Q were eligible to participate (*n* = 201). The participants (*n* = 108) represented all four years of the medical school and included different nationalities. They were 56% female (*n* = 61) and 44% male (*n* = 47). Participants who consented to the study were sent an email link for 2 questionnaires available through Qualtrics XM: (1) the Revised 2-Factor Study Process Questionnaire (R-SPQ-2F) and (2) the “How Medical Students Choose” Questionnaire (HMSCQ).

### Instruments

We measured students’ approaches to learning with the R-SPQ-2F developed by Biggs et al. [11] in 2001. The R-SPQ-2F features 20 5-point Likert items that frame students’ approaches to learning within the dynamic 3P model. *Presage* items measure student characteristics like prior knowledge and ability, *process* items evaluate ongoing approaches to learning, and *product* items gauge the influence of teaching context. The R-SPQ-2F displays results on a matrix of 2 main scales—Deep Approach (DA) and Surface Approach (SA)—and five subscales, Deep Motive (DM), Deep Strategy (DS), Surface Motive (SM), and Surface Strategy (SS) [11]. *Motive* describes students’ intentions for engaging in learning tasks—including fear of failure, achievement, and intrinsic interest—while *strategy* involves how students go about completing learning tasks; for instance, using rote memorization or relating new knowledge to previous learning [11, 23]. The R-SPQ-2F offers critical insight into the ways students perceive and engage learning tasks in specific teaching contexts. However, while the R-SPQ-2F has the flexibility to split DA and SA into further subscales, our analysis only computed students’ scores for DA and SA.

We constructed the “How Medical Students Choose” Questionnaire (HMSCQ) to record demographic information, measure students’ specialty interests, and explore their subjective perceptions of specialty characteristics. We adapted it from a questionnaire developed by Wright et al. [24], which aimed to determine the factors influencing medical students’ choice of primary care specialties in rural Canada. Our adaptation reflects the Qatari context by including specialties identified by Qatari policymakers as national priorities, such as primary care and medical research. The HMSCQ features 43 5-point Likert items with 6 underlying constructs representing students’ perceptions of specialties: (1) medical lifestyle, (2) social orientation, (3) prestige, (4) hospital orientation, (5) role model, and (6) varied scope of practice. The HMSCQ also collected demographic information such as gender, age, educational attainment, parental educational attainment, and previous experience with the health care system.

### Data analysis

We analyzed the results using IBM SPSS v27 and AMOS Graphics, and the data are reliable according to the following Cronbach’s alpha values: (1) HMSCQ (0.905), (2) R-SPQ-2F DA (0.781), and (3) R-SPQ-2F SA (0.785). The WCM-Q Institutional Review Board (IRB) approved the study after ethical review of the study protocol (Reference Number 18 − 00009).

## Results

**Fig. 1:**
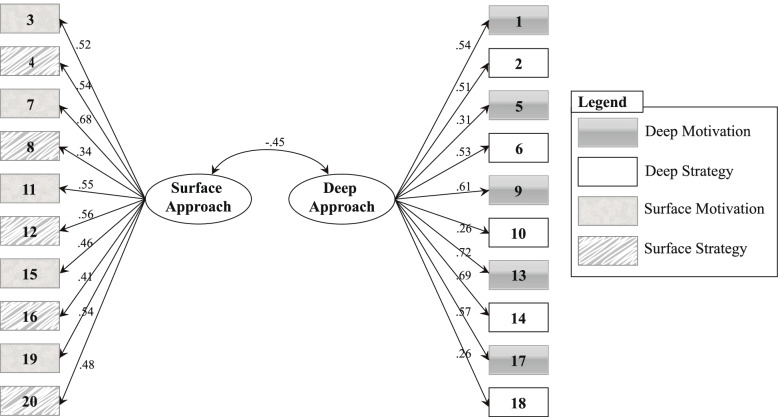
Two-factor measurement model of the R-SPQ-2 F with four subscales

Our first research question aimed to verify how well the R-SPQ-2F fits the Qatari context. We employed structural equation modeling (SEM) to confirm the underlying 2-factorial structure for the 20-item R-SPQ-2F. This verified the instrument’s construct validity with data collected from WCM-Q medical students. The model shown in Fig. 1 includes the items for the main scales *a priori* [11]. The measurement model generated from the R-SPQ-2F items—10 for DA and 10 for SA—shows factor loadings for the 2 subscales. The item-factor loading ranged from 0.26 (items 10 and 18) to 0.72 (item 13). The initial chi-square statistic result was χ^2^ = 217.24, *df* = 163, *p* < 0.003, which indicates the model’s testability. We then evaluated the model for the fitness of indices, including the comparative fit index (CFI) and the root-mean-square error of approximation (RMSEA) [25]. A small RMSEA of 0.49 and a large CFI of 0.92 suggest that the model cohesively fits the data [26, 27].

Once the instrument’s fit was established, we aimed to understand how medical students in Qatar approach learning tasks. Table 1 presents descriptive statistics of student responses to the DA and SA subscales. It also presents the results of a one-way ANOVA illustrating variances in students’ approaches to learning between different year levels (Years 1–4). **(**Table 1**)** Medical students reported varying levels of preference for deep approaches to learning, but surface approaches remained constant. Year 3 reported the greatest preference for deep learning (3.06) while Year 2 reported the lowest (2.55). The eta-squared values (effect sizes) were 0.07 for deep approaches and 0.02 for surface approaches. The range of variance in coefficient values for some items measuring deep approach—for instance, items 10 (“I test myself on important topics until I understand them completely”) and 18 (“I make a point of looking at most of the suggested readings that go with the lectures”)—may explain differences in the perception of deep approaches to learning between various medical cohorts. The *F* of 2.701 shows significant differences in deep approaches toward medical learning at WCM-Q. Students’ preference for deep approaches indicate preparation for and engagement in PBL as part of their medical training.

**Table 1:** Approach to learning: Descriptive statistics and one-way ANOVA results

Scale *(20 items)*	Med 1 (*n* = 36)		Med 2 (*n* = 20)		Med 3 (*n* = 19)		Med 4 (*n* = 33)		*F*	Sig. (*p < 0.05)
	Mean	Std. Dev.	Mean	Std. Dev.	Mean	Std. Dev.	Mean	Std. Dev.		
DA *(10 items)*	2.89	0.68	2.55	0.45	3.06	0.63	2.70	0.67	2.701	0.049*
SA *(10 items)*	2.51	0.79	2.35	0.57	2.27	0.58	2.34	0.65	0.612	0.61

We were also interested in how student characteristics influence approach to learning. First, we examined the relationship between gender and approach to learning. Table 2 indicates that men (2.97) prefer deep approaches more than women (2.67), with a statistically significant difference between the groups. On the other hand, no significant gender differences were identified for surface approaches.

**Table 2:** Approach to learning and gender: Descriptive statistics and one-way ANOVA results

Scale *(20 items)*	Female (*n* = 61)		Male (*n* = 47)		*F*	Sig. (*p < 0.05)
	Mean	Std. Dev.	Mean	Std. Dev.		
DA *(10 items)*	2.67	0.66	2.97	0.60	5.895	0.017*
SA *(10 items)*	2.30	0.67	2.49	0.68	2.054	0.155

Next, we explored the relationship between educational attainment and approach to learning. Table 3 illustrates this relationship using one-way ANOVA analysis. As shown in the table, no statistically significant differences arose for deep approaches, but the mean scores for surface approaches ranged between 2.33 and 2.95. The effect sizes calculated using eta-squared were 0.04 for DA and 0.09 for SA. Medical students with non-science qualifications preferred deep approaches to learning more than their peers did. Mean scores for preference for surface approaches ranged from 1.10 (Bachelor of Arts) to 2.95 (Masters).

**Table 3:** Approach to learning and educational qualification prior to enrollment in medical education

Scale	High School (*n* = 83)		Bachelor of Science (*n* = 12)		Bachelor of Arts (*n* = 2)		Masters (*n* = 2)		Other (*n* = 9)		*F*	Sig. (*p* < 0.05)
	Mean	Std. Dev.	Mean	Std. Dev.	Mean	Std. Dev.	Mean	Std. Dev.	Mean	Std. Dev.		
DA *(10 items)*	2.75	0.63	2.88	0.66	3.60	0.14	3.20	0.57	2.86	0.80	1.15	0.337
SA *(10 items)*	2.39	0.65	2.33	0.65	1.10	0.65	2.95	0.65	2.57	0.65	2.49	0.048

We also explored the relationship between prior experience with health care and approach to learning. Prior experience with health care included activities like previous employment, volunteer experience, suffering from a major health problem, or having a family member who suffers from a major health problem. Remarkably, 1 student cited other reasons and 15 students did not specify any prior experiences. Table 4 shows no statistically significant differences between deep and surface approaches; however, based on mean scores, previous employment (3.17), volunteer experience (2.81), and having a family member who suffers from a major health problem (2.86) correlated with greater preference for deep approaches. None of these factors influenced preference for surface approaches. The eta-squared values in Table 4 show the proportion of the variance of students’ deep approach that is explained by their prior experience. A value of 0.044 indicates small to medium effect size [28].

**Table 4:** Students’ approach to learning and experience prior to enrollment in medical education: Descriptive statistics and one-way ANOVA results

Subscales *(20 items)*	Employment (*n* = 6)		Volunteering (*n* = 58)		Major health problem (*n* = 4)		Family member (*n* = 24) was a patient		Other (*n* = 1)		None (*n* = 15)		*F*	Sig.	Eta	Eta squared
	Mean	Std. Dev.	Mean	Std. Dev.	Mean	Std. Dev.	Mean	Std. Dev.	Mean	Std. Dev.	Mean	Std. Dev.				
DA *(10 items)*	3.167	0.779	2.807	0.696	2.675	0.4924	2.863	0.535	2.8	1.97	2.54	0.6	0.939	0.459	0.21	0.044
SA *(10 items)*	2.317	0.578	2.298	0.74	2.675	0.4031	2.433	0.659	2.4	1.70	2.6	0.515	0.672	0.646	0.179	0.032

## Discussion

 This study explored the factorial structure of the R-SPQ-2F for medical students in Qatar; examined how student characteristics—specifically gender, educational attainment, and prior experience with health care—correlated with preference for deep or surface approaches to learning; and considered the implications of students’ preference for deep approaches for their involvement in inquiry-based learning activities like PBL. First, the statistical validation reaffirms the R-SPQ-2F as a reliable and valid instrument, supporting previous studies from different national and cultural contexts [18, 19, 26, 29]. The reiteration of acceptable fit indices (as outlined in) from the 2-factor model supports the theoretical interpretability of the subscales for understanding medical students’ approaches to learning in Qatar.

Although medical students at WCM-Q prefer deep approaches overall, our data show that preferences vary across year level. This finding resonates with studies from Australia, Saudi Arabia, Turkey, and Ghana, which suggest that medical students typically prefer deep approaches to learning [17, 19–21]. However, the intensity of preference fluctuates because of contextual factors like curriculum and assessment [17, 21]. Interestingly, preference for surface approaches decreased as students progressed through the medical program, which supports previous findings that deep approaches to learning grow with greater maturity [12, 17, 30]. WCM-Q’s new curriculum may work to sustain deeper approaches to learning, as it features diverse assessments, varied experiences, and student-centered teaching methods. Students’ strong overall preference for deep approaches complements the growing use of PBL, as shown in previous research [17, 21], and indicates students’ preparation for and engagement in PBL. We attribute the slight dip in students’ preference for deep approaches during the second year of medical school to their preparation for the USMLE Step 1 exam, an 8-hour multiple-choice licensing examination. Students spend March and April of their second year preparing for this important evaluation, which encourages surface approaches to learning. During this period, just before clerkships begin, students lose focus on their intrinsic motivation for medicine and concentrate on achieving an excellent result instead. This upholds the findings of research into high-stakes testing in schools, which show that high-stakes testing narrows the curriculum, diminishes teaching and learning quality, and encourages surface approaches to learning [30, 31]. It also resonates with the findings of Tetik et al. [21], who identified a slight reduction in deep approach for second-year students at 2 of the 3 medical schools they examined. The USMLE Step 1 during Year 2 and involvement in off-campus hospital clerkships during Year 3 may also explain the relatively lower response rates for these year levels, though studies have shown that response rates for online surveys trend low [16, 32].

As stated in the 3P model, student characteristics like gender, educational attainment, and previous experience interact with the teaching context to influence preference for deep approaches to learning. Regarding gender, we found a statistically significant difference between men and women, with men more likely to prefer deep approaches. This contrasts with earlier studies, which found either no difference or stronger preference among women [17, 30, 33, 34]. We attribute this difference to cultural and contextual factors. Abu-Hilal et al. [34] suggest that differential socialization for boys and girls in Gulf Arab societies influences self-concept, goal orientation, and academic achievement. They argue that girls in Gulf Arab cultures gain a self-improving orientation aimed at correcting limitations while boys develop a self-enhancing orientation characterized by an “unrealistically positive self-opinion” [34]. Studies show that girls in Gulf Arab countries academically outperform boys, and Abu-Hilal et al. ascribe their higher academic achievement to greater extrinsic motivation and an improvement-oriented self-concept [34, 35]. Girls in Gulf Arab societies face greater pressure to achieve because of more limited educational and career options, and this “achievement anxiety” inhibits deep approaches to learning. However, Abu-Hilal et al. [34] observe that Gulf Arab culture and society are rapidly transforming, so additional research is needed to validate this interpretation.

Medical students’ educational attainment and prior experiences with health care mildly correlated with preferences for deep approaches to learning. Medical students with high-school qualifications showed a lower preference for deep approaches than their peers with post-secondary qualifications; however, this could also be attributed to greater maturity. Remarkably, while only 2 participants had non-science backgrounds, they showed lower preference for surface approaches to learning. That being said, several studies have shown that the greatest single predictor of success in medical school and residency is academic achievement, irrespective of undergraduate discipline [36–39]. Finally, prior experience with health care—previous employment in a health care field, medical volunteer experiences, and having an ill family member—contributed to the preference for deep approaches to learning. This suggests that early exposure to medicine and healthcare organizations might enhance intrinsic motivation for learning about medicine.

### Limitations

While a key strength of the study was its contribution to further validating the R-SPQ-2F in a new cultural context, we overlooked other similar questionnaires. In addition, we conducted the study at a single institution, a transnational American medical school in Qatar. This unique cultural and environmental context limits the generalizability of results to other settings. Finally, as participation in the study was voluntary, the sample could be non-representative. However, we made tried to recruit a mix of participants that represented the national, social, and cultural groups present at WCM-Q.

## Conclusions

The results from this study reaffirmed the reliability and validity of the R-SPQ-2F as a tool for measuring students’ deep and surface approaches to learning, which supports the findings of earlier studies. The R-SPQ-2F’s 2-factor structure showed acceptable fit indices, confirming the instrument’s utility for understanding the learning approaches of medical students in Qatar. As this was the first time such a study has been conducted in Qatar, our results are exploratory. First, we found that medical students in Qatar prefer deep approaches to learning, and that their reliance on surface approaches gradually decreases. This finding is consistent with previous studies. We suggest that curricular changes, including increased use of PBL, have complemented and enhanced the preferred deep approaches to learning. However, contextual factors like the high-stakes USMLE examination for second-year students may trigger temporary declines in preference for deep approaches. Second, our finding that men prefer deep approaches more than women contrasted with earlier studies, which found either no difference or greater preference for deep approaches among women. This difference may arise from Qatar’s cultural context, but we need further research to confirm this interpretation. Finally, our finding that educational attainment and prior experiences with health care correlate to preference for deep approaches may be due to the opportunities these experiences offer for learning about medicine and health care more broadly. Mature, academically experienced students and those with significant early exposure to medicine are more likely to find medicine intrinsically interesting. Future research should investigate other aspects of WCM-Q’s teaching context, including the introduction of a mandatory research module, and how these enhance or reduce preference for deep approaches. These findings may help medical education leaders in Qatar improve pedagogical practices and student outcomes in the future.

## Data Availability

The datasets used and/or analyzed during the current study are available from the corresponding author on reasonable request.

## Abbreviations

CFI: Comparative Fit Index
DA: Deep Approach
DM: Deep Motive
DS: Deep Strategy
GPA: Grade Point Average
HMSCQ: “How Medical Students Choose” Questionnaire
PBL: Problem-Based Learning
RMSEA: Root-Mean-Square Error of Approximation
R-SPQ-2F: Revised Study Process Questionnaire - 2 Factor
SA: Surface Approach
SEM: Structural Equation Modeling
SM: Surface Motive
SRMR: Standardized Root-Mean-Squared Residual
SS: Surface Strategy
USMLE: United States Medical Licensing Examination
WCM-Q: Weill Cornell Medicine-Qatar
